# The African Development Corridors Database: a new tool to assess the impacts of infrastructure investments

**DOI:** 10.1038/s41597-022-01771-y

**Published:** 2022-11-09

**Authors:** Jessica P. R. Thorn, Diego Juffe Bignoli, Ben Mwangi, Robert A. Marchant

**Affiliations:** 1 School of Geography and Sustainable Development, University of St Andrews, St Andrews, United Kingdom; 2 York Institute of Tropical Ecosystems, Department of Environment and Geography, University of York, York, United Kingdom; 3 African Climate and Development Initiative, University of Cape Town, Cape Town, South Africa; 4 Department of Environmental Sciences, University of Namibia, Windhoek, Namibia; 5 United Nations Environment Programme World Conservation Monitoring Centre, Cambridge, UK; 6 Durrell Institute of Conservation and Ecology, University of Kent, Canterbury, UK; 7 Institute of Climate Change and Adaptation, University of Nairobi, Nairobi, Kenya

**Keywords:** Environmental impact, Developing world, Biodiversity, Sustainability, Industry

## Abstract

The large-scale expansion of built infrastructure is profoundly reshaping the geographies of Africa, generating lock-in patterns of development for future generations. Understanding the impact of these massive investments can allow development opportunities to be maximised and therefore be critical for attaining the United Nations’ Sustainable Development Goals and African Union’s Agenda 2063 aims. However, until now information on the types, scope, and timing of investments, their evolution and spatial-temporal impact was dispersed amongst various agencies. We developed a database of 79 development corridors across Africa, synthesizing data from multiple sources covering 184 projects on railways, wet and dry ports, pipelines, airports, techno-cities, and industrial parks. The georeferenced interlinked tabular and spatial database includes 22 attributes. We expect this database will improve coordination, efficiency, monitoring, oversight, strategic planning, transparency, and risk assessments, among other uses for investment banks, governments, impact assessment practitioners, communities, conservationists, economists, and regional economic bodies.

## Abstract (summary)

**Table:** 

Measurement(s)	infrastructure projects
Technology Type(s)	Geographic information systems
Factor Type(s)	Project code • Project name • Corridor name • Infrastructure/development type • Status • Country • Region or province • Description • Launch year • USD amount (million) min • USD amount (million) max • Amount description • Distance (km) min • Distance (km) max • GIS distance • Area • Supplier or recipient of goods or services • Key beneficiaries • Commodities traded or transported • Name of donors or financiers • Amount funded (USD million) per donor type • Type of major donor/financiers
Sample Characteristic - Environment	Anthropognenic
Sample Characteristic - Location	Africa

## Background

Investment in infrastructure globally is at an all-time high^1,2^ while in recent decades Africa has seen accelerating construction of complex networks^3^. This exponential increase in investment is situated against a long history of under-investment^4^, being home to some of the fasting growing economies^5^, having an urban population estimated to grow by over 60% by 2060^6,7^, and a growing appetite from domestic and foreign direct investors. Although the numbers are contested, Africa only has about 1% of the world’s railways, about a third of the rural population has access to roads, less than 5% has irrigated agriculture, while only 40% has electricity, and there are few direct flights connecting parts of the continent^8,9^. Consequently, infrastructure needs are high: between 2013–2017 the annual funding was USD 77 billion, double the annual average between 2000–2006, which is estimated to further double to USD 150 billion by 2025^10^. With the promises of the Belt and Road Initiative, the G7 Build Back Better World Partnership and the European Union’s Global Gateway, such investments are likely to increase.

Although there are multiple definitions (e.g.^11–13^), infrastructure corridors generally deliver services such as energy, water, waste management, transport, and telecommunications; and often lead to spatial development between rural peripheries and urban growth poles^1,14,15^. Infrastructure corridors become development corridors when larger, often transnational, and linear, geographical areas are targeted for domestic and international investment^16^. Development corridors typically go beyond individual investments, have political-economic weighting, are attached to Master Planning processes, are positioned as flagship initiatives^17^, and ideally, underpinned by Strategic Environmental Assessments^16,18^.

Such development corridors have the potential to offer development gains but also open extensive areas of land to new environmental pressures and are not without social-economic challenges^19^. On the one hand, development corridors are considered as “dreamscapes of modernity” and critical elements for promoting transformational change, sustainable development, and synergistic climate change mitigation and adaptation^13,20^. On the other, development corridors can lead to significant biodiversity loss, habitat fragmentation^3,16^, pollution, spread invasive species^21^, increase illegal logging, poaching and fires^22^, severely affect river deltas and coastal and marine ecosystems^23^, and consume large volumes of greenhouse gas intensive products such as steel and cement^24^. Beyond environmental impacts, development corridors can widen inequalities between stakeholders who are not party to the planning process but affected by it^11^, lead to transformations in local livelihoods^25^, increase adjacent communities’ exposure to natural hazards, leave an unsustainable burden of debt^1^, and are often fraught with anxieties of social contestation^13,26^. As infrastructure enters more remote areas, the significance increases^27^.

There have been previous attempts to map corridors - not least the work of the Programme for Infrastructure Development in Africa Information Centre^28^, the G20’s Global Infrastructure Hub^29^, the European African Infrastructure Trust Fund Information Centre^30^, the Tripartite Transport and Transit Facilitation Programme^31^, the African Transport Policy Programme, the African Union’s NEPAD Africa Infrastructure Database, the World Bank’s Public Private Partnership Knowledge Lab, and various private sector portals tracking traffic, logistics and trade. However, these have not been integrated in a continental database to understand impacts on people and biodiversity. Contributing to this, is a lack of fine-grained, disaggregated data that can be systematically compared across space and time. Moreover, development corridors rarely have clearly demarcated boundaries, titles, stages of evolution, and durations^16^. Limited GIS capacity and understanding of assumptions in data preparations for analyses feed into a lack of data standardisation which yields non-comparable results^32^.

The clear lack of data on impacts furthermore leads to the following challenges^16,20,27,33^: (1) governance, coordination, and transparency difficulties; (2) inefficiencies in operations and maintenance; (3) scale mismatches (e.g., national interests outweigh local welfare or international connectivity); (4) trade-offs across sectoral domains (e.g., economic interests outweigh land conversion); (5) contradicting, scattered and ambiguous information allows manipulation depending on priorities, diverse interests, and outcomes; (6) inadequate or rare implementation of best practice mitigation measures; and (7) politically polarised discourse in the media, which can reflect popular attitudes and shape opinion. These are only some of the challenges that are realised at the local level (e.g., for environmental practitioners tasked with undertaking timely Environmental Impact or Strategic Environmental Assessments), and at a global level (e.g., where international assessments such as recent the Global Environmental Outlook^34^, Global Infrastructure Outlook^9^ or the International Panel on Biodiversity and Ecosystem Services^35^ may rely on inaccurate information). Governments and private sector players need to step up to prepare, plan, and manage development corridors and their constituent projects with a new level of rigour and robustness.

Here, we present a new comprehensive African Development Corridors Database, and document where, when, and how development corridors have evolved across the continent. Our work builds on and offers a wider scope and level of detail than previous efforts by research of mapping development corridors in Africa.

Overall, our dataset increases:

1. the *scope* of possible analyses, featuring spatial, temporal, and sectoral resolutions of development corridor networks that were so far not publicly available,
2. the *comparability* of these analyses based on a standardised and comparable dataset, and
3. *reproducibility* as we provide a common input data framework using a data standard and provide open access to the data.

In using this database, we emphasise the necessity of future regional and subregional analyses to ensure data is kept up-to-date and accounts for the dynamic nature of investments. Such analyses should be combined with capacity building of users to analyse these data with other complementary knowledge products, such as carbon, water, biodiversity, risk of ecosystem collapse, capacities for conservation response, proxies for resilience, inequality, electricity access, or land tenure^36^. Clear guidelines of how to standardise data among regions will be necessary, as well as making explicit assumptions underlying the data^32^.

## Methods

**Fig. 1:**
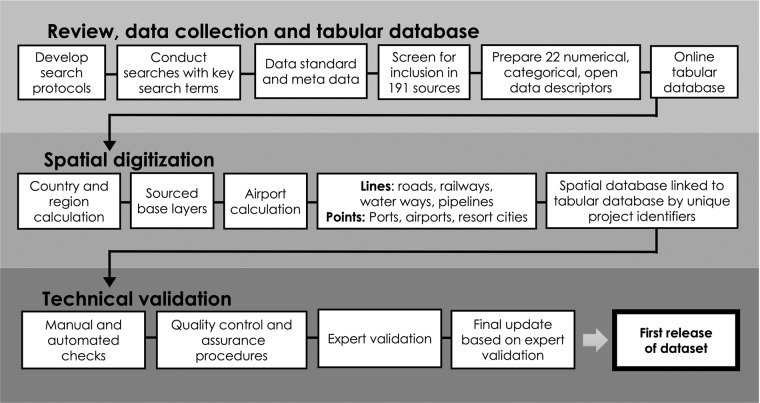
Workflow diagram of development corridors review, mapping, and technical validation procedure to compile the African Development Corridors database.

The methodology was set in three main stages: first, a global review involving the searching and screening for data, second, the manual digitisation of spatial features, spatial temporal distribution, and volume calculation procedures, and third, technical validation. The main steps for each stage are summarised in Fig. 1.

### Search strategy

Standard methods were used to access, appraise, and synthesise peer reviewed and grey literature about infrastructure projects within development corridors in Africa. We developed a search protocol to ensure rigor, objectivity, verifiable procedures, and clarity of the study design^37^. The search strategy was established through discussions researchers and stakeholders during eleven months of fieldwork in Tanzania and Kenya between 2018 and 2019, and consultations with the executive board of scientific experts in the Development Corridors Partnership  (https://developmentcorridors.org). The Development Corridor Partnership is a collaborative capacity building research programme in Kenya, Tanzania, China, and the UK involving universities, development corridors agencies and NGOs. Three reviewers conducted searches and screening over 18 months between the 28^th^ of January 2020 and 19^th^ May 2021.

### Sources of publications and key search terms

We combined a multitude of data sources (*n* = 190), and ran searches on websites of corridor authorities, regional economic bodies, national government bodies, private companies, INGOs, international and national media houses, research groups, public private partnerships, regional associations, research groups, and multilateral agencies (see Supplementary Table 1 for a summary of these data sources). In addition, we searched multilateral development banks across the world (e.g., African Development Bank, Asian Development Bank, European Central Bank World Bank, Islamic Development Bank), which have approved funding for infrastructure development projects with wider socio-economic developmental benefits. We also searched infrastructure databases, including e-platforms for information and knowledge sharing on infrastructure development in Africa. Four key e-journals whose topic areas closely aligned with the research focus were hand searched, including Geographical Review, Economic Geography, African Studies Quarterly and International Journal of Applied Engineering Research. We further contacted researchers involved in past and ongoing projects to source data, such as African Ecological Futures and the Global Infrastructure Mapping and Modelling Project.

After testing the key search terms on 20 development corridors to confirm that the search strings captured relevant literature, and balanced specificity and sensitivity^38^, the final string consisted of: “transport corridor *“, “economic corridor *“, “development corridor *“, “infrastructure corridor*“, “resource corridor*“, and “growth corridor*“. These terms were applied to all databases and sources for all African countries, subject to their individual search requirements, following. We used Boolean operator terms and wildcards, i.e., a character that can be used to substitute for other character(s) in a string, connected search terms, which were disaggregated using truncation (“*” in most databases).

### Inclusion criteria

We only included infrastructure projects for which we could see a clear connection to a development corridor programme as defined above. We divided infrastructure within development corridors into the following types: airports; dry ports; wet ports; electricity transmission lines; freight railways; industrial parks; passenger and freight railways; pipelines (oil); pipelines (water); resort cities; and roads. We included electricity transmission lines only when multiple countries had partnered to develop the infrastructure to enhance trade. Waterways included all forms of transport of goods and people traversing water, including catamarans and large ships. We included wet ports that cater for large shipping vessels, as well as dryland inland ports. We included international airports which are written into development strategic planning or policy documents, are international hubs which allow for the inflow and outflow of traded goods and services in development corridors or are physically connected or near development corridors included in our database. We excluded dams, domestic electricity transmission lines, small ferry links, freshwater inland ports, local airports, agricultural corridors, bridges (unless part of roads in a development corridor), and other forms of infrastructure not connected to development corridors.

Searches were completed for 53 countries in continental Africa. Islands of Comoros, Mauritius, Reunion, Mayotte and São Tomé and Principe were excluded because we did not find development corridors present in these areas. English, Swahili, Portuguese, and French sources were reviewed, but we did not include other languages due to the skillset of the review team. We expect that these languages captured most key documents regarding development corridors in Africa, and suggest future iterations include languages such as Arabic, Yoruba and Hausa. Research articles, reviews, and reports were included, while book chapters, conference proceedings and graduate theses were excluded. There was no time limitation on the searches.

### Spatial digitisation of corridors

Mapping of the spatial features of the African Development Corridors Database was made using ArcGIS Desktop 10.8.1 and ArcGIS Pro 2.5.2. Each development corridor project was identified with a unique project code, to which all tabular and spatial parameters were associated, recorded as point or line features. We sourced available base layers using the results of Laurance *et al*.^3^, Open Street Maps (2021)^39^, the World Food Programme GeoNode Global Airports dataset (2021)^40^ including international airports or airports that were within a buffer of 40 km to nearby development corridors, and data from partners linked to the Development Corridors Partnership. We also looked for maps in reports and websites and digitised them. Approximately 79.1% (*n* = 140 projects) were fully mapped, while the remaining 44 (23.9%) were unmapped.

For those development corridors that were mapped, we identified which countries boundaries development corridors features intersected with. For those corridors which were not mapped, we identified countries based on the literature. We used the country name as per ISO 3166 standard (2021)^41^ and the country boundaries as per the Level 1 from Global Administrative Boundaries dataset version 3.6 (2018)^42^. All distances were computed for the line features using the calculate geometry tool in ArcGIS. Before measuring the geodesic distance, we ran the dissolve tool to remove the overlaps and therefore avoid overestimating the distances when two different corridors share the same portion of a given road or railway. We then calculated the total sum of the length of corridors for each country. We did not include berths, quays, and wharfs due to wide range in sizes of these structures, depending on the country, ship size and study context.

## Data Records

The African Development Corridors database is publicly available. The visualisation of the database that can be explored interactively here: https://dcp-unep-wcmc.opendata.arcgis.com/. The data is deposited in the Dryad Digital Repository referenced as Thorn, J. P.R., Mwangi, B.; Juffe Bignoli, D., The African Development Corridors Database, Dryad, Dataset, 10.5061/dryad.9kd51c5hw (2022)^43^. The final data were compiled into an online Master database spreadsheet, using the project code data as the merging attribute of the spatial and tabular database (AfricanDevelopmentCorridorsDatabase2022.csv). The African Development Corridor Database is presented as a GeoPackage file (.gpkg) and ESRI file Geodatabase (.gdb) composed by line and point feature datasets with the 22 associated attributes for all mapped corridors, a table with corridors that could not be mapped (also with the attributes), and a table with all sources consulted for each project code.

We created a data standard to ensure a systematic and standardised data collection (Supplementary Table 2). Each data record in the database represents a project within a development corridor. To group all projects within the same development corridor we used a unique identifier composed by three letters that identified the corridor plus a number unique for each project or record. For example, the Lamu port project in Kenya within the Lamu Port South Sudan Ethiopia Transport Corridor (LAPSSET) was represented as LAP000. In this corridor we identified 20 projects, from LAP0001 which is the Lamu Port to LAP0020 which is the Isiolo-Lokichar-Lodwar-Nadapal Highway in Kenya. In addition to the unique identifier for each project, the data standard includes data attributes that provide detailed information about each project. Table 1 describes the attributes included in the database. Supplementary Table 3 summarises the 79 corridors included in the database.

**Table 1:** List of the attributes included in the African Development Corridors Database.

No	Attributes	Format	Description	Validation requirement
1	Project code	Code	Unique identifier of development corridor project	Three letters, four numbers
2	Project name	Text	Commonly used name to refer to a project within a development corridor.	Number of characters <100
3	Corridor name	Text	Specific name of the development corridor often formed by multiple projects.	Number of characters <100
4	Infrastructure/development type	Text	Dry port; electricity transmission; freight railway; industrial park; passenger and freight railways; oil pipelines; water pipelines; port; resort city; road; waterway.	From list
5	Status	Text	In progress; on hold; operational; planned; upgrade	From list
6	Country	Text	Country(s) where the development corridor project is located.	From list (ISO 3166 codes)
7	Region or province	Text	Region(s) where the development project is located	From list
8	Description	Text	Short narrative of main objectives, functions, and features of the project of a development corridor	Free text
9	Launch year	Number	Year of completion and inauguration of the project	Year (must be in the past)
10	USD amount (million) min	USD	Investment cost minimum	Numerical digits
11	USD amount (million) max	USD	Investment cost maximum	Numerical digits
12	Amount description	Text	Description of what the minimum and maximum amount refers to: partial project cost; total project cost; or cost of upgrade	Free text
13	Distance (km) min	km	Minimum linear distance of corridor element as reported in the sources reviewed	Numerical digits
14	Distance (km) max	km	Maximum linear distance of corridor element as reported in the sources reviewed	Numerical digits
15	GIS distance	km	Linear distance calculated in ArcGIS	Numerical digits
16	Area	km^2^	Total area of corridor element in square kilometres as reported in the sources reviewed if data is available for all infrastructure except for ports and airports which we are represented as points	Numerical digits
17	Supplier or recipient of goods or services	Text	Specifies whether the project is predominantly a net supplier or recipient of goods or services	From list
18	Key beneficiaries	Text	Describes who are the beneficiaries of the corridor	From list
19	Commodities traded or transported	Text	Lists key commodities traded, exchanged, or transported within the corridor.	From list
20	Name of donors or financiers	Text	Name of entity or entities funding the amount specified in the amount field.	From list
21	Amount funded (USD million) per donor type	USD	Specifies the amount funded for each donor.	Numerical digits
22	Type of major donor/financiers	Text	Entity or entities funding each amount: international development agencies; national development agencies; multilateral banks; national governments; national governments; public private partnerships; private companies; regional development banks; regional economic communities.	From list

### Infrastructure types and status of development corridors in Africa

**Fig. 2:**
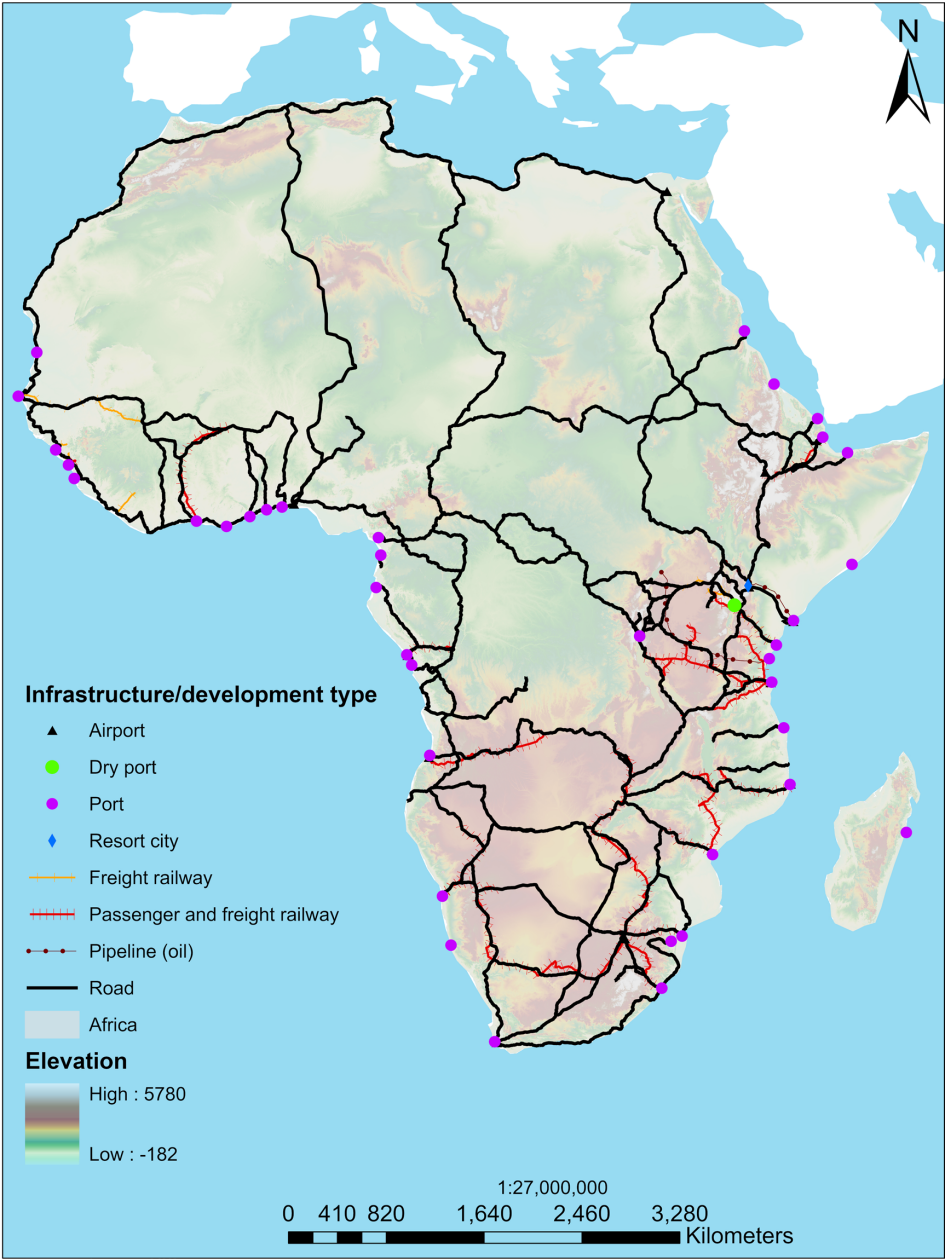
Map showing the distribution of all the development corridors included in the African Development Corridors Database and their infrastructure type.

**Fig. 3:**
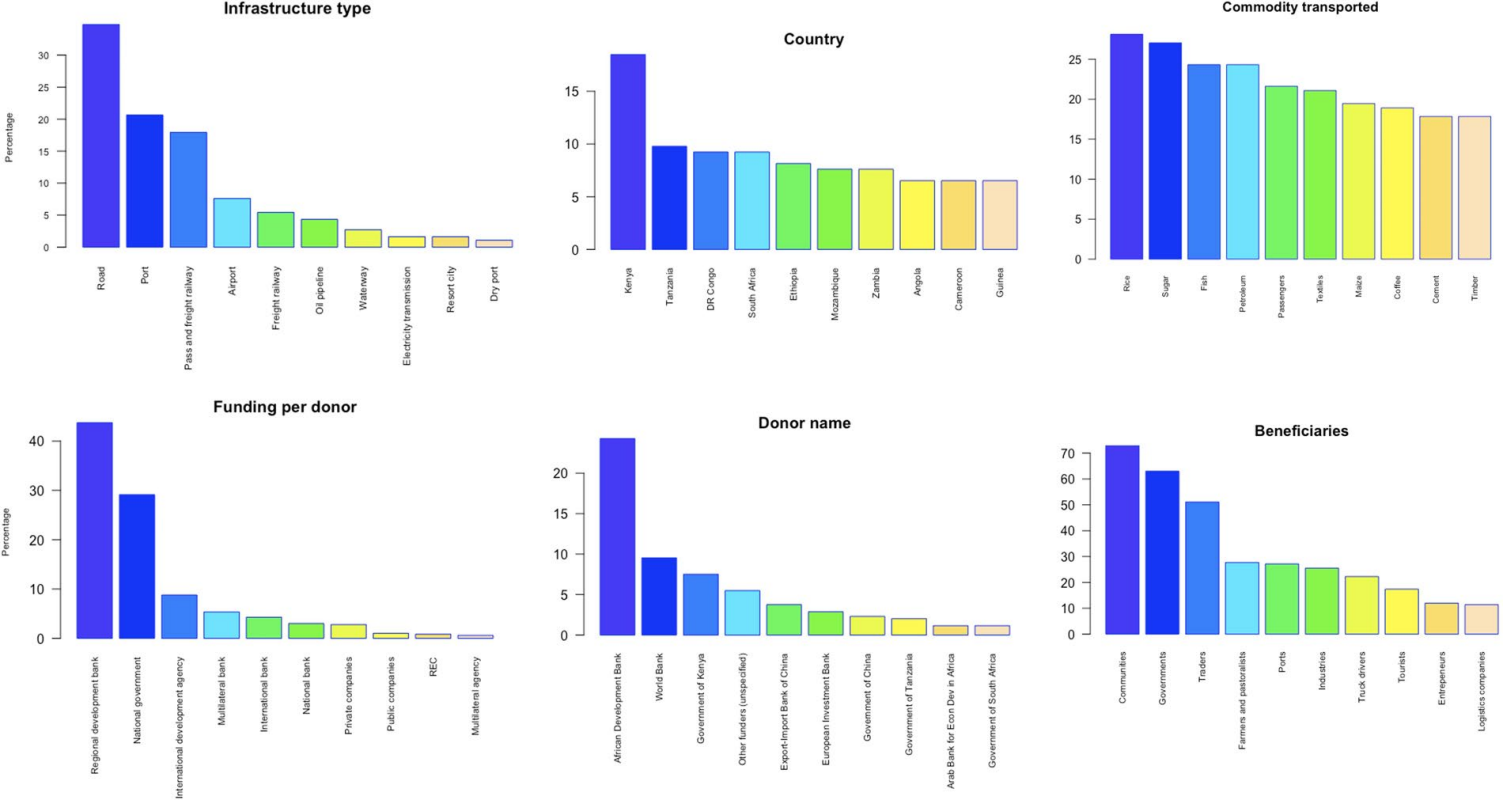
Subset of highest frequencies of key attributes captured in the database.

The data consists of a total of 79 corridors consisting of 184 projects (Fig. 2). Of the 12 infrastructure types, the most predominant form of infrastructure in Africa’s development corridors is roads (*n* = 64, 34.8%), followed by wet ports (*n* = 38, 20.7%), passenger and freight railways (*n* = 33, 17.9%), and airports (*n* = 14, 7.6%). Fewer resort cities, electricity transmission lines, dry ports, industrial parks, and water pipelines comprise development corridors (all ≤ *n* = 3, 1.6%) (Fig. 3). We acknowledge our study might not include many infrastructure developments that are components projects of larger programmes but are not yet labelled as corridors. A total of 107 (58.7%) projects are operational, 35 (19%) are in progress, 25 (13.6%) are planned, 25 (13.9%) are being upgraded, and 2(1%) are on hold.

### Spatial distribution

The linear distance of development corridors in Africa is 122,294 km – which approximates to three times the Earth’s circumference, with an average of 1703.84 ± 213.19 km (mean, SE), ranging from 4–11,141 km. In terms of number of projects per country, Kenya has the most projects (*n* = 34, 18.5%), followed by Tanzania (*n* = 18, 9.8%), South Africa and Democratic Republic of the Congo (*n* = 17, 9.2% ea.), Ethiopia (*n* = 15, 8.2%), Mozambique and Zambia (*n* = 14, 7.6%), Angola, Uganda, Guinea and Cameroon (*n* = 12, 6.5%), Namibia (*n* = 11, 6.0%), Republic of Congo (*n* = 10, 5.4%), Burundi and Chad (*n* = 9, 4.9%), Malawi, Senegal, and Zimbabwe (*n* = 8, 4.4%), and Burkina Faso and Ghana (*n* = 7, 3.8%). Due to differences in the frequency and quality that countries publish data on infrastructure and development corridor investments, coverage may be lower for some regions, or some periods (i.e., recent, or further in the past).

### Investments in development corridors

Adjusting for inflation, the total investment of development corridors that is captured in the database ranges between USD 547.29–658.62 billion. The average cost of a corridor ranges between USD 3.46 ± 1.92 billion and USD 4.17 ± 2.04 billion. This is a notable sum, considering the average GDP in sub-Saharan Africa is USD 1.48 billion^44^. The most expensive development corridor project is the first of the nine Trans African Highway projects at USD 78.20 billion (when adjusted for inflation) – comprising transcontinental roads across Africa. We were able to capture the budget (or at least a proportion of the budget) for 84.7% of all projects.

### Temporal evolution of growth of development corridors

**Fig. 4:**
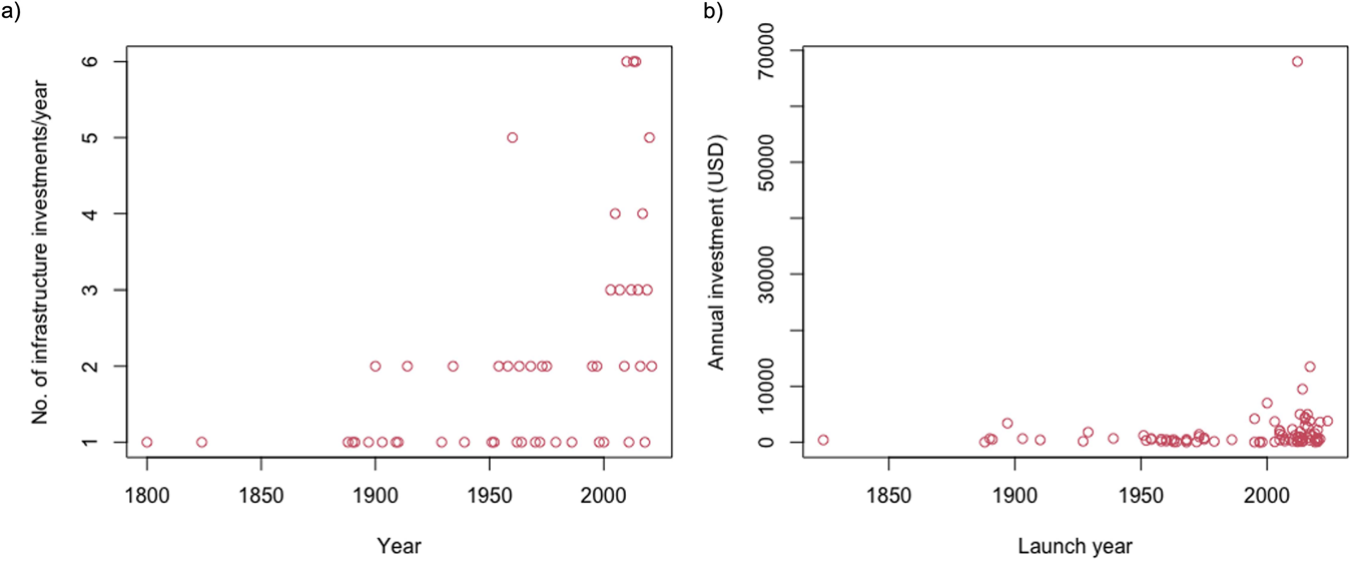
(**a**) Temporal evolution of investment in development corridors in Africa. (**b**) Annual investments per annum in development corridors in Africa (USD maximum, before adjusting for inflation).

Investments started in the 1800s and have increased exponentially (Fig. 4). Over a fifty-year period, the greatest number of investments took place between 1950 and 2000. Spikes in investments occurred particularly around 1900, which was when there was a wave of new imperialism across the continent, followed in the 1960s when many countries across sub-Saharan Africa gained independence. The third spike in investment was in the last decade, particularly since 2013, when we have seen rapid growth in foreign direct investment in Africa under initiatives such as the Belt and Road Initiative. According to the Ernst and Young Africa Attractiveness Survey (2019)^45^, the largest foreign direct investment (in terms of capital) between 2014–2018 came from China (USD 72,235 million), France (USD 34,172 million), USA (USD 30,885 million), the United Arab Emirates (USD 25,278 million) and the United Kingdom (USD 17,768 million).

### Donors that are funding development corridors

Across Africa, regional development banks invested the most in development corridors (30.8%), with the African Development Bank funding the majority (24.3%) of all projects. Outside of Africa, the regional development banks that invested in the most projects are the Export-Import Bank of China (*n* = 13, 3.8%), the European Investment Bank (*n* = 10, 2.8%) and the Arab Bank for Economic Development in Africa (*n* = 4, 1.2% ea.). National governments funded about 29.8% of all projects. The Government of Kenya funded the most projects (*n* = 26; 7.5%), followed by the Governments of Tanzania (*n* = 7, 2.0%) and South Africa (*n* = 4, 1.2%). Multilateral banks funded 10.9% of projects - mostly from the World Bank (*n* = 33, 9.54%) and a few from the International Finance Corporation (*n* = 4, 1.6%). The international development community funded only 6.1% - of which the OPEC Fund for International Development funded four projects. Private companies continue to invest in a small percentage of development corridors (3.5%), but this is higher than national banks that invest in 3.2%. Regional Economic Community bodies have invested in 15 (4.8%) of all 184 projects. The average number of donors per corridor ranged from one to 12.

### Weighting of investments by donor type

In terms capital funded per donor type, Regional Development Banks invested the most (totalling USD 30.72 billion), followed by national governments (USD 20.45 billion). The figure then drops substantially to international development agencies (USD6.17 billion) and multilateral banks (USD 3.76 billion). These results are limited by the fact that we were only able to capture the amount funded delineated by donor type for 22.58% (or USD 70.24 billion) of the minimum of all investments (USD 311.14 billion) before adjusting for inflation.

### Commodities transported

A total of 147 commodities were captured. The top twenty commodities traded were rice (*n* = 52, 28.7% of all projects), sugar (27.0%), fish and petroleum (24.3% ea.), passengers (21.6%), textiles (21.1%), maize (19.5%), coffee (18.9%), cement and timber (17.8% ea.) followed by cotton, crude petroleum, vehicle spare parts, beverages, copper, fruit, fertilisers, gold, pharmaceutical products, and tobacco.

### Beneficiaries and net supplier or receiver

Approximately 213 different beneficiaries were identified – predominantly local communities (*n* = 134 of projects, 72.8%), followed by national and local governments (63.0%), traders (51.1%), agricultural farmers and livestock producers (27.7%), ports (27.2%), industries (25.5%), truck drivers (22.3%), tourists (17.4%), entrepreneurs (12.0%), and logistics companies (11.4%). Almost all (89.1%) of corridors are net receivers and suppliers of commodities, while only 13 (7.1%) are net suppliers and seven are net receivers (3.8%).

## Technical Validation

The database represents a collation of publicly available data published by official sources. Therefore, we conducted a critical appraisal to ensure the database represents an accurate record of the official data. We used the following four main strategies.

First, all data collected, whether obtained from official channels or from third-party repositories of official data, was subject to initial manual verification when it was added to our database for the first time^46^. Five quality criteria were used to assess whether sources were of sufficient quality for inclusion (following^47,48^): (1) data collection methods, both qualitative and quantitative, are thoroughly explained, (2) methods are clear and replicable, (3) key terms were well defined, (4) conclusions are logically derived, and (5) conclusions supported by presented evidence. We only used international and national media sources which are well-reputed when no other data source could be identified. This process was iterative, including reassessments of the validity and robustness of the data sources, and checks of updated website links.

Second, we applied a range of quality control and quality assurance procedures to verify, collate, and standardise both the manual and automated data added to the database. We continually ran manual checks throughout the workflow to check for invalid data. Ambiguities were discussed in regular weekly meetings, and secondary reviewers verified decisions. For other metrics, we compared data across all different sources available and used a range to determine if our lowest and highest values are reasonable. We monitored changes in development corridor status in each country throughout the data extraction process. To ensure data integrity was maintained throughout the project, and limit data transcription errors and eliminate naming inconsistencies during manual input, we designed dropdown lists of predefined values, which were regularly modified to list new possibilities. We furthermore limited the number of ‘free text’ fields. Completeness of data entry was mandated by users, i.e., the user could not continue with the workflow unless all input fields were completed, following Martin *et al*.^49^. Alongside eliminating naming inconsistencies, input validation against specified parameters was associated with each field. A summary of these validation requirements is presented in Table 1 (e.g., Project code - Unique identifier of development corridor project – three letters, four numbers).

For economic cost and account for annual inflation, where the launch year of the project was available, we adjusted the investment value using the Consumer Price Index^50^. This index represents changes in prices of all goods and services purchased for consumption by urban households from 1913 to the present. For years before 1913, we used the 1913 value, and for years beyond 2021, we used the 2021 value. For years that did not have information on the launch year, we did not adjust the investment value. We acknowledge that this inflation rate does not account for changing costs over time (e.g., operation and maintenance), nor the fact inflation rates fluctuate from the start to the completion of a given project. We also acknowledge these data are limited by the financial data publicly available and may not include information about costs such as compliance requirements, permitting processes, contracts, or easements.

Third, visual checks were carried out. Since some of the routes were outdated, routes were verified and updated using Open Street Maps which is updated daily. We then verified these layers using an ESRI World Imagery map^51^. Spatial and tabular datasets were reviewed and evaluated against selected studies that have mapped development corridors in Africa^3^. Where the relative position of the position of the development corridor projects and previous mapping efforts were not aligned, differences were discussed between authors and with regional experts until a consensus of the updated route or position was reached. When a project within a particular corridor overlapped in space with another project or corridor, we kept these routes as separate records in the database to allow independent future analysis per development corridor or project. Prior to the date when the African Development Corridors Database was made available online, it was thoroughly tested and verified following protocols developed by the authors in Ravilious *et al*.^52^.

Fourth, we conducted an expert review over one month. This involved getting feedback from 50 secondary reviewers. These experts worked across Africa with a deep knowledge in infrastructure developments, representing a diversity of countries and organisations. We drew from the networks of the authors and the Development Corridors Partnership advisory board that had been developed between 2017–2021. Snowball sampling also allowed us to contact referrals to recruit a wider array of secondary reviewers. We then shared the review call on email, social media, and websites. We provided access to a dynamic online version of the database where the spatial and tabular data could be interrogated interactively. We captured reviewers’ comments in a standardised feedback form. We obtained a 40% rate of response for the expert review which resulted in a new updated version of the database (Table 2).

**Table 2:** Experts from across Africa who contributed to the technical review assessment.

Institution type	Institution
Multilateral agency	United Nations Environment Programme Sustainable Infrastructure Partnership
	United Nations Environment Programme Economic Research Unit
	United Nations Environment Programme Gender and Safeguards
Development Bank	African Development Bank - Safeguards and Compliance Department
	World Bank
	International Finance Corporation
Private companies	Constellation Group
	Independent consultant
	Southern Agricultural Growth Corridor of Tanzania
	Biotope
	Business Continuity Planning
	CR Attorneys
	Jabu
	WSP Environmental Consulting
Organisations	Worldwide Fund for Nature Africa
	Worldwide Fund for Nature Cameroon
	Worldwide Fund for Nature Madagascar
	Worldwide Fund for Nature Uganda
	Worldwide Fund for Nature Kenya
	Worldwide Fund for Nature Tanzania
	Worldwide Fund for Nature Norway
	African Conference on Linear Infrastructure Ecology
	Wildlife Conservation Society
	Endangered Wildlife Trust
	Grevy’s Zebra Trust
	Birdlife
	International Union of the Conservation of NatureThematic Group on Impact Mitigation and Ecological Compensation
Research institutions	University of Stellenbosch
	London School of Economics
	Yale University
	ETH Zurich
	Centre for Science and Technology Innovations
	UNIBAS - Coloniality of Infrastructure conference chair
	University of York
	South African National Biodiversity Institute

## Usage Notes

We provide a spatially-explicit database of development corridors in Africa. This newly compiled dataset builds upon previous efforts to map corridors at a continental level (e.g., Laurance *et al*.^3^). Of particular use is the wider scope, level of detail and rigour, including 22 data attributes. This provides a valuable resource for improving our quantifiable understanding of large-scale impacts of infrastructural development corridors on biodiversity, protected areas, hydrological processes, and ecosystem services, their spatial dynamics, and their interactions with climate, land use and global environmental change. To assess the potential risk of impact on conservation areas, analyses can be undertaken to evaluate the spatial relationships between this dataset and others. For example, one can determine how development corridors overlap spatially with protected areas, by assessing the intersection of the corridor line feature dataset with the World Database on Protected Areas and Other Effective Area Based Conservation Measures (WDPA/OECM)^53^ and apply different buffer choices to corridor lines to estimate the geographic extent of that risk (e.g.^3^).

Additionally, the inclusion of the cost of corridors within the database can contribute to our understanding of economic losses when stranded infrastructure assets are written down, devalued, or converted to liabilities due to growing intensity and frequency of extreme events, or resource scarcity. The inclusion of different infrastructure types and their distribution can help inform integrated regional in-country strategic planning – allowing planners to consider infrastructure more easily as a system of systems across all stages of infrastructure’s lifecycle. When overlaid with other variables, data can be used for environmental impact assessments or strategic environmental assessments to determine how where proposed developments may minimise harm and maximise socio-economic and environmental co-benefits over time (e.g., considering fossil fuel lock in) and space (e.g., densification *vs*. sprawl) and intensity of the development corridor use under different scenarios (e.g., demographic trends, consumer demand). Data can be used to inform decisions of how to mobilise financial and non-financial resources, build capacity, improve coordination, and facilitate policy formulation and improve transparency on reporting and monitoring. Data can moreover help network traditionally separate firms and industries to utilise resources for newly built, upgraded, or retrofitted infrastructure. As such, we provide an example of how the database can assess impacts (i.e., on protected areas), but leave the manipulation and interpretation of the data up to the discretion of the user.

The database can be used under the license of Creative Commons (CC BY), meaning that the work can be distributed, adapted, and built upon with acknowledgement of authors. There are several ways to access and download the information contained in the database – with options in *.CSV, *.KML, Shapefiles or GEOJSON formats. Detailed information about the variables in the database can be found in Supplementary Table 2, which corresponds to the *.CSV files in the data portal.

The recent and ongoing negotiations of global development targets have brought attention to improving the accessibility and sustainability of infrastructure to the fore and how this relates to other interrelated goals. For instance, the Sustainable Development Goal 9 aims to “*build resilient infrastructure, promote inclusive and sustainable industrialisation and foster innovation*”, while SDG 11 aims to “*make cities and human settlements inclusive, safe, resilient and sustainable*”^54^. Meanwhile, the Convention on Biological Diversity’s post-2020 Global Biodiversity Framework calls for 30% of globally important land for biodiversity and its contributions for people to be effectively and equitably managed, well-connected, and integrated into the wider landscape^55^. We therefore hope this database is one step to develop greater clarity on the potential impacts of development corridors on social-ecological outcomes at large spatial-temporal scales and take action to ensure synergistic delivery of benefits^56^.

However, given there are limits to regional mapping approaches, our database must be complemented by contextually rich, empirical assessments and situated knowledge and practice that build regional understandings from the ground up^57^. To ensure the ongoing reliability of the data - we envision the database will be updated as more data is digitised and made available - if resources allow. Further contributions, regular updates or analysis based on, and complementing the African Development Corridors Database will be welcome.

## Supplementary information

Supplementary material Thorn et al 2022

## Data Availability

No customised code was produced to prepare or analyse the dataset.
