# Supplementary material for: The African Development Corridors Database: a new tool to assess the impacts of infrastructure investments
Source: Sci Data. 2022 Nov 9;9:679. doi: 10.1038/s41597-022-01771-y (PMC9646820; doi:10.1038/s41597-022-01771-y)
Supplement: Supplementary file 1 — Supplementary material Thorn et al 2022 [file 41597_2022_1771_MOESM1_ESM.pdf]

Supplementary material: The African Development Corridors Database:

a new tool to assess the impacts of infrastructure investments

Jessica P. R. Thorn[1,2,3,4]; Diego Juffe Bignoli [5,6]; Ben Mwangi[7]; Robert A. Marchant[3]

[1] School of Geography and Sustainable Development, University of St Andrews, United Kingdom

[2] York Institute of Tropical Ecosystems, Department of Environment and Geography, University of York, United Kingdom

[3] African Climate and Development Initiative, University of Cape Town, South Africa

[4] Department of Environmental Sciences, University of Namibia, Windhoek, Namibia

[5] United Nations Environment Programme World Conservation Monitoring Centre, UK

[6] Durrell Institute of Conservation and Ecology, University of Kent, UK

[7] Institute of Climate Change and Adaptation, University of Nairobi, Kenya

Corresponding author: Jessica P. R. Thorn (jprt1@st-andrews.ac.uk)

Table of Contents

SUPPLEMENTARY TABLE 1. SOURCES OF PUBLICATIONS. 2

SUPPLEMENTARY TABLE 2. DATA STANDARD.. 6

SUPPLEMENTARY TABLE 3. LIST OF THE 79 DEVELOPMENT CORRIDORS INCLUDED IN THE AFRICAN DEVELOPMENT CORRIDORS DATABASE. 9

Supplementary Table 1. Sources of publications.

Some of the development authorities' names may change over time, or evolve to other regional bodies in the future, while the links were correct on 1 November 2022.

Category	Source	Link
**Data portals and e-platforms**	Programme for Infrastructure Development in Africa	https://www.au-pida.org/
	Global Infrastructure Hub	https://www.gihub.org/
	Global Infrastructure Connectivity Alliance	https://www.gica.global/maps
	NEPAD Africa Infrastructure Database	https://aid.nepad.org/index.php/auth/register
	The IUCN Water Knowledge Platform	http://www.waterandnature.org/
	Vital Signs	http://vitalsigns.org/
	OpenStreetMap	https://wiki.openstreetmap.org/wiki/Main_Page
	Vessels Tracker	https://www.vesseltracker.com/en
	Tripartite Transport and Transit Facilitation Programme	https://tttfp.org
	African Transport Policy Programme	https://www.ssatp.org
	PPP Knowledge Lab	https://library.pppknowledgelab.org/
	The Observatory of Economic Complex	https://oec.world/en
	World Port Source	http://www.worldportsource.com
	Southern African Research and Documentation Source	https://www.sardc.net/en/
	Early Warning System Database	https://ewsdata.rightsindevelopment.org
	FleetMon Maritime Vessels and Ports Database	https://www.fleetmon.com/
	Logistics Capacity Assessments	https://dlca.logcluster.org/display/public/DLCA/LCA+Homepage
	UK Foreign, Commonwealth and Development Office Dev Tracker	https://devtracker.fcdo.gov.uk/regions/298/
	Find a Port	https://www.findaport.com/
	4AllPorts	http://www.4allports.com/
	Swiss Arab Entrepreneurs Platform	https://www.saentrepreneurs.ch/
	Maritime Services Directory	https://ports.co.za/directory_front.php
	Marine Traffic	https://www.marinetraffic.com/en
	SKYbrary Electronic Repository	https://www.skybrary.aero
	Seaports Information for The Maritime Community	https://seaport.homestead.com/
	Construction Review Online	https://constructionreviewonline.com
	Market Research	https://www.marketresearch.com
	Airport Technology	https://www.airport-technology.com
	Ship Technology	https://www.ship-technology.com
	Port Technology	https://www.porttechnology.org
	Railway Technology	https://www.railway-technology.com
	Hydrocarbons Technology	https://www.hydrocarbons-technology.com/projects/
	Road Traffic Technology	https://www.roadtraffic-technology.com/
	European African Infrastructure Trust Fund Information Centre	https://www.eu-africa-infrastructure-tf.net/infocentre/index.htm
	Railway Pro	https://www.railwaypro.com/wp/
	Dangerous Roads	https://www.dangerousroads.org/africa/
	CW Group Research	https://www.cwgrp.com/research/
	Cameroon Trade Hub	https://www.cameroontradehub.cm/documentation/bibliotheque/1/en
**Regional economic communities**	Economic Community of West African States	https://www.ecowas.int
	East African Community	https://www.eac.int
	African Union Development Agency NEPAD	https://www.nepad.org/project
	African Union	https://au.int/en/resources/filter
	European Commission	https://www.eeas.europa.eu/eeas/publications_en
	Union for the Mediterranean	https://ufmsecretariat.org/info-center/
	Southern African Development Community	https://www.sadc.int/documents-publications/

**Multilateral development banks**	African Development Bank Data Portal	https://projectsportal.afdb.org/dataportal/VProject/list
	African Development Banks African Water Facility	https://www.africanwaterfacility.org/
	European Investment Bank	https://www.eib.org/en/publications/index.htm
	World Bank Africa Infrastructure National Database	https://databank.worldbank.org/source/africa-infrastructure:-national-data
	World Bank International Finance Corporation	https://www.ifc.org/
	World Bank Private Participation in Infrastructure Database	https://ppi.worldbank.org/en/ppi
	Islamic Development Bank	https://www.isdb.org
	International Monetary Fund	https://www.elibrary.imf.org
**Corridor authorities**	East African Crude Oil Pipeline	https://eacop.com
	Lamu Port Corridor Development Authority	https://www.lapsset.go.ke/
	Maputo Corridor Logistics Initiative	https://www.mcli.co.za/maputo-development-corridor/
	Trans African Concessions	https://tracn4.co.za/
	Cross Border Road Transport Agency	https://www.cbrta.co.za/
	Northern Corridor and Transport Coordination Authority	http://www.ttcanc.org/page.php?id=26
	North South Corridor Africa	http://en.reingex.com/North-South-Corridor-Africa.shtml
	Tripoli Windhoek Trans African Highway	http://en.reingex.com/Tripoli-Windhoek-Corridor.shtml
	Trans Africa Pipeline	https://transafricapipeline.org/
	Central Corridor Transit Facilitation Agency	https://centralcorridor-ttfa.org
	Walvis Bay Corridor Group	http://www.wbcg.com.na/
	Standard Gauge Railway Uganda	https://www.sgr.go.ug/
	Southern Agricultural Growth Corridor of Tanzania	https://sagcot.co.tz/
**National authorities**	Ghana Ports and Harbours Authority	https://ghanaports.gov.gh
	Beira Corridor	https://beiracorridor.org/
	Berbera Port Authority	https://www.berberaseaport.net
	Port Cotonou	http://www.portcotonou.com
	Togo Port Authority	https://www.togo-port.net/
	Abu Dhabi Ports	https://www.adports.ae/
	Kenya Ports Authority	https://www.kpa.co.ke/
	Kenya Railways Corporation	http://krc.co.ke/
	NamPort Authority	https://www.namport.com.na/
	Government of Malawi Roads Authority	https://www.ra.org.mw/
	Cameroon Ministry of Economy, Planning and Regional Development	https://www.minepat.gov.cm/index.php?lang=en
	Kenyan Ministry of Lands and Physical Planning	https://www.landportal.org/organization/ministry-lands-and-physical-planning
	South African Limpopo Economic Development Agency	https://www.lieda.co.za/Wordpress/
	Tanzania Zambia Mafuta (TAZAMA) Pipelines Limited	https://projectsportal.afdb.org/dataportal/VProject/show/P-ZM-DE0-001
	Tanzania Ports Authority	https://www.ports.go.tz/
	City of Cape Town Invest Cape Town	https://www.investcapetown.com/
	South Africa Parliamentary Monitoring Group	https://pmg.org.za/
	Republic of Guinea Ministry of Mines and Geology	https://mines.gov.gn/en/priorities/infrastructure/
**International organisations**	Japanese International Cooperation Agency	https://openjicareport.jica.go.jp
	Japanese Embassy in Eritrea	http://www.eritreaembassy-japan.org/
	Japanese Ministry of Food and Agriculture	https://www.mofa.go.jp/region/africa/
	United States Agency for International Development	https://www.usaid.gov/reports-and-data
	United States International Trade Administration	https://www.trade.gov/trade-data-analysis
	United States Embassy and Consulate in Nigeria	https://ng.usembassy.gov/
	Chinese Embassy in Tanzania	http://tz.china-embassy.org/eng/
	Netherlands Ministry of Foreign Affairs	https://www.rvo.nl/
	Agence Française de Développement	https://www.afd.fr/en
	German Cooperation Agency	https://www.giz.de/en/html/index.html

	Organisation for Economic Commission for Development	https://www.oecd.org/
	United Nations Conference on Trade and Development	https://unctad.org/publications
	World Food Programme Geonode Global Airports	https://geonode.wfp.org
**Regional associations**	Southern African Railways Association	https://sararail.org
	Africa Ports	https://africaports.co.za
	Trademark East Africa	https://www.trademarkea.com/publications/
	Centre for Aviation	https://centreforaviation.com/data
	Macau Hub Science Promotion	https://macauhub.com.mo
	Nile Equatorial Lakes Subsidiary Action Program	https://nelsap.nilebasin.org/index.php/en/
**Journals**	Geographical Review	https://onlinelibrary.wiley.com/journal/19310846
	Economic Geography	https://academic.oup.com/joeg
	African Studies Quarterly	https://asq.africa.ufl.edu/
	International Journal of Applied Engineering Research	https://www.ripublication.com/ijaer.htm
**Public private partnerships**	Borderless Alliance West Africa	https://www.borderlesswa.com
	EKN	https://www.ekn.se/en/
	The Public Private Infrastructure Advisory Facility	https://ppiaf.org/
**Research groups**	International Growth Centre	https://www.theigc.org
	Development Corridors Partnership	https://developmentcorridors.org
	Macleki	https://macleki.org
	Oxford Institute for Energy Studies	https://www.oxfordenergy.org/library/
**INGOs**	Fahamu Networks for Social Justice	http://www.fahamu.org/about-us/
	Global Security	https://www.globalsecurity.org
	Bank Track	https://www.banktrack.org
	Trade Law Centre (tralec)	https://www.tralac.org/about.html
**Private companies**	Vallis	https://vallis-group.com/research-2/
	Maputo Port Development Company	https://www.portmaputo.com
	INON Africa	https://www.inonafrica.com/
	Bollore Transport and Logistics	https://www.bollore-transport-logistics.com/en/
	Saana Consulting	http://www.saana.com
	COWI	https://www.cowi.com/solutions/infrastructure/
	Artelia Passion and Solutions	https://www.arteliagroup.com/en/
	Inras Lackner	https://www.inros-lackner.de/en/projects
	Searates	https://www.searates.com
	EGIS Engineering Services Group	https://www.egis-group.com
	Proger company	http://www.proger.it/en/we/
	Limak Group of Companies	http://www.limak.com.tr/
	Geoscience Ireland	https://www.geoscience.ie/
	Nathan Incorporated Consulting	https://www.nathaninc.com/
	AgDevCo	https://www.agdevco.com
	Aurecon Group	https://www.aurecongroup.com/projects/
	Novonor	https://www.novonor.com.br/en/home
**International media houses**	The Economist Intelligence Unit	http://www.eiu.com/default.aspx
	Reuters	https://www.reuters.com
	Financial Times	https://www.ft.com/
	Rail Journal	https://www.railjournal.com/africa/
	Railway Gazette International	https://www.railwaygazette.com/
	International Railway Journal	https://www.railjournal.com/africa
	Global Construction Review	http://www.globalconstructionreview.com
	African Mining Market	https://africanminingmarket.com/
	Business Daily Africa	https://www.businessdailyafrica.com/
	World Highways	https://www.worldhighways.com/
	Infrastructure News	https://infrastructurenews.co.za/

	The New Humanitarian	https://www.thenewhumanitarian.org/
	ThinkGeoEnergy	https://www.thinkgeoenergy.com/
	The North African Post	https://northafricapost.com/
	How we made it Africa	https://www.howwemadeitinafrica.com/
	The Exchange: Africas Investment Gateway	https://theexchange.africa/
	All Africa	https://allafrica.com/
	Journal of Commerce Online	https://www.joc.com/
	Duta	https://indianexpress.com/about/duta/
	Reliefweb	https://reliefweb.int
	China Africa	http://www.chinafrica.cn
	Simple Flying	https://simpleflying.com/category/africa/
	Tefnanet News	https://tesfanews.net/
	Engineering news	https://www.engineeringnews.co.za
	Devex	https://www.devex.com/
	African Law and Business	https://iclg.com/alb
	Jeune Afrique BTP Infrastructures	https://www.jeuneafrique.com/rubriques/btp-infrastructures/
	Pana Press	https://www.panapress.com/actualite-lang2.html
	Daily Southern and East African Tourism Update	https://www.tourismupdate.co.za
	The Africa Report	https://www.theafricareport.com/
**National media houses**	Club of Mozambique	https://clubofmozambique.com/
	Daily Monitor Uganda	https://www.monitor.co.ug/
	The Independent Uganda	https://www.independent.co.ug/
	Construction Kenya	https://www.constructionkenya.com/
	Kenya Engineer	https://www.kenyaengineer.co.ke/
	The Star Kenya	https://www.the-star.co.ke
	IOL	https://www.iol.co.za/business-report/international
	Krugersdorp News	https://krugersdorpnews.co.za
	Cameroon Business	https://www.cameroonbusinesstoday.cm/
	CGTN	https://www.cgtn.com
	Xinhuanet News	https://www.borderlesswa.com
	The East African	https://www.theeastafrican.co.ke
	China Daily	http://global.chinadaily.com.cn
	Lusaka Times	https://www.lusakatimes.com
	Business in Cameroon	https://www.businessincameroon.com
	Cameroon Tribune	https://www.cameroon-tribune.cm
	The New Times Rwanda	http://www.newtimes.co.rw
	Tanzania Invest	https://www.tanzaniainvest.com/
	Brana Press Ethiopia	http://www.branapress.com
	Ethiopia Semoegna	https://semonegna.com/
	Agencia Angola Press	https://www.angop.ao
	Anadolu Agency	https://www.aa.com.tr/en/africa/
**Other datasets**	Laurance et al. (2015)	https://www.sciencedirect.com/science/article/pii/S0960982215013093

Supplementary Table 2. Data standard.

Field	Type	Description	Accepted values	Example
Project code	Code	The code comprises a combination of the corridor name, comprising three initials and four digits. Use consecutive numbers as new projects within the corridor are added.	3 letters for code<br>4 numbers for project number	SGR0001
Project name	Text	Commonly used name to refer to a project within a development corridor. Roads and highways are used interchangeably by applying the term which was used most frequently in documentation.	Project name (100 characters)	Mombasa-Nairobi Standard Gauge Railway Project
Corridor name	Text	Specific name of the development corridor often formed by multiple projects. For some corridors which have the same name in two contexts, we added a contextual description (e.g., the North South Corridor in Southern and Eastern Africa, and the North South Corridor in Egypt). Where there was not consensus of the title of corridor, multiple names are given, or we use the main cities of origin and destination. If there was an alternative name for the corridor, this was included in the description.	Development corridor name (Max 100 characters)	Standard Gauge Railway
Infrastructure/development type	Text	Infrastructure types included roads, passenger and freight railways, oil and water pipelines, resort cities and industrial parks. We included electricity transmission lines when multiple counties have partnered to develop the infrastructure to enhance trade. Waterways included all forms of transport of goods and people including catamarans and large ships. Wet ports were included that cater for large shipping vessels, and dryland inland ports. International airports were included which are written into development strategic planning or policy documents, are international hubs which allow for the inflow and outflow of goods and services in the development corridors and improve trade and communications or are physically connected to development corridors in our database. We excluded dams, domestic electricity transmission lines, small ferry links, freshwater inland ports, local airport, agricultural corridors, bridges (unless part of roads in a development corridor), and other forms of infrastructure not connected to development corridors	Airport<br>Dry port<br>Electricity transmission<br>Freight railway<br>Industrial parks<br>Passenger and freight railway<br>Pipeline (oil)<br>Pipeline (water)<br>Port<br>Resort city<br>Road<br>Waterway<br>Other	Passenger and freight railway
Status	Status	We assessed the five stages of corridor development. Planning included a new corridor that is still at the conceptual stages and yet to be implemented. In progress refers to a corridor that is being constructed. Operational means the main elements of the corridor are partially (c. 75%) or fully completed in a way that allows activity. Upgrading refers to an existing corridor whose infrastructure is being enhanced or significantly updated (e.g., expanding a road from single to a dual carriage). This only applied for major rehabilitations. On hold refers to a corridor that has stopped or temporarily stalled. Where sections of a project within a corridor had multiple stages, such as a road that was upgraded in one section, while it was operational in another section, we selected the status on the most advanced stage. We acknowledge this data would be highly temporally bound and users of the database should review the latest update of the database.	In progress<br>On hold<br>Operational<br>Planned<br>Upgrade<br>No information (NI)	Operational
Country	Text	Country(s) where the project of the development corridor is located. Different values are separated by ";" and countries should be listed alphabetically.	Country name as per ISO 3166 standard (https://www.iso.org/iso-3166-country-codes.html)<br>No information	Kenya; Tanzania
Region or province	Text	Region or province where the corridor is located. Different values are separated by ";". As we calculated this in ArcGIS 2.5.2, where there was no spatial information, or the project is planned, we state “NI”.	Region of province name<br>No information	Kajiado; Kwale; Machakos; Makueni; Mombasa; Nairobi; Taita Taveta

Description	Text	Short narrative of main objectives, functions, and features of the project or corridor.	A short summary of the project details (254 characters)<br>No information (NI)	The Mombasa–Nairobi Standard Gauge Railway connects the port city of Mombasa to Kenya's capital city Nairobi.
Launch year	Year	Launch year refers to the completion and inauguration of the project. If there were multiple dates in terms of the upgrades of a project (e.g., a railway constructed in the 1900s and then upgraded in 2012), we used the original construction date. If the project experienced a significant upgrade, then the year of renovation was indicated, and the construction year (if available) was included in the comments. Where we included a future year, we acknowledge that this is the expected date recorded but there may be potential delays. If the status of a corridor was an upgrade, then the launch year refers to when the upgrade started. NI was used if no information could be found. NA was used when planned projects had not yet been launched.	Numeric value<br>No information (NI)<br>Not Applicable (NA)	2007
USD amount (million) Minimum	Number	Project budget (minimum) in USD	Numeric value<br>No information (NI)	3000
USD amount (million) Maximum	Number	Project budget (maximum) in USD	Numeric value<br>No information (NI)	2200
Amount description	Number	Description of what the minimum and maximum amount refer to. It could be the budget for a whole corridor, a corridor project, or a section of the project.	Cost of upgrade<br>Partial project cost<br>Total project cost	Partial project cost
Distance (km) Minimum	Number	Linear distance of corridor element in kilometres as reported in the sources reviewed (if applicable).	Numeric value<br>No information (NI)<br>Not Applicable (NA)	445
Distance (km) Maximum	Number	Linear distance of corridor element in kilometres as reported in the sources reviewed (if applicable).	Numeric value<br>No information (NI)<br>Not Applicable (NA)	550
GIS distance	Km	Linear distance calculated in ArcGIS.	Numerical value	1000
Area (km^2)	Text	Total area of corridor element in square kilometres as reported in the sources reviewed. This applies to all infrastructure except for ports and airports which are represented as points and polygons based on the available data.	Numeric value<br>No information (NI)	220
Supplier or recipient of goods or services	Text	Specifies whether the project is a supplier or recipient of goods or services.	Predominantly net supplier<br>Predominantly net recipient<br>Both<br>No information (NI)	Predominantly net supplier
Key beneficiaries	Text	Beneficiaries of the corridor. Values are separated by different values using ";".	Beneficiaries of the corridor<br>No information	Farmers; private companies
Commodities traded or transported	Text	Key commodities traded by this project. Values are separated by different values using ";".	Commodities traded, exchanged, or transported within the corridor<br>No information (NI)	Sugar, crude petroleum oil
Name of donors or financiers	Text	Name of entity or entities funding the amount specified in the amount field. Values for different donors are separated by “;".	Name of the bank	African Development Bank; World Bank
Amount funded (USD million) per donor type	USD	Funded amount for each donor in USD million. Values for different donors are separated by ";".	Numeric value<br>No information (NI)	60%;40%

Type of major donors or financiers	Text	Type of entity or entities funding each amount. Separate by different values using “;". International development agencies are defined as international agencies that work to provide financial, technical, or other support to low- and middle-income countries. National development agencies are defined as national government agencies that work to provide for low- and middle-income countries. Multilateral banks are defined as financial institutions that are characterised by two or more countries working together to provide financial and technical assistance for development in low- and middle-income countries. Finance is allocated through low-interest concessional loans or grants. National governments are defined as institutions and leaders that represent a political authority with members from more than one political party that controls a nation. Private companies are defined as businesses that are a segment of a national economy that are owned, managed, and controlled by individuals and organisations seeking to generate profit. Public-private partnerships are defined as cooperative arrangements between two or more public and private sector actors that work together to complete a project and/or to provide services to the public. Regional Development Banks are defined as financial institutions composed of memberships from several countries in a specified region that provide financial and technical assistance for development in low- and middle-income countries. Regional Economic Communities are defined as regional groupings of African states, formed with the purpose to facilitate regional economic integration between members of the individual regions and through the African Economic Community.	International development agency<br>Multilateral banks<br>National development agency<br>National government<br>Private companies<br>Public private partnership<br>Regional development bank<br>Regional economic community<br>No information (NI)	Regional Development bank; Multilateral banks

Supplementary Table 3. List of the 79 development corridors included in the African Development Corridors Database.

No.	Project code	Corridor name	Infrastructure type	Status	Country
1	AAC0001	Assab-Addis Ababa Corridor	Road and port	Operational	Eritrea; Ethiopia
2	ABC0001	Abidjan-Bamako Corridor	Road	Upgrade	Côte d'Ivoire; Mali
3	ADC0001	Addis-Djibouti Corridor (or Ethiopia-Djibouti Corridor)	Passenger and freight railway; Port; Road; Airport	Upgrade; Operational	Djibouti; Ethiopia
4	ALC0001	Abidjan-Lagos Corridor	Road	In progress	Benin; Côte d'Ivoire; Ghana; Nigeria; Togo
5	AOC0001	Abidjan-Ouagadougou Corridor	Road; Passenger and freight railway	Operational; Upgrade	Burkina Faso; Côte d'Ivoire
6	BAA0001	Berbera-Addis Ababa Corridor (or Berbera Corridor)	Road; Port	Operational	Ethiopia; Somaliland
7	BCC0001	Bas Congo Corridor	Road	Operational	Democratic Republic of Congo
8	BLC0001	Beira Lobito Corridor (or Lobito Corridor)	Pipeline (oil); Port; Passenger and freight railway; Industrial parks; Airport	In progress; Operational	Angola; Democratic Republic of Congo; Mozambique; Zambia; Zimbabwe;
9	BSC0001	Bamako – San Pedro Corridor (or San Pedro-Bamako Corridor)	Road	Operational	Côte d'Ivoire; Mali
10	BVH0001	Beitbridge-Victoria Falls-Harare Corridor	Road	In progress	Zimbabwe
11	BYC0001	Brazzaville-Yaounde Corridor (Brazzaville-Douala Corridor)	Road, Port	In progress; Operational; Upgrade	Cameroon; Republic of the Congo
12	CCO0001	Chad-Cameroon Oil Corridor	Pipeline (oil)	Operational	Cameroon; Chad
13	CDC0001	Central Development Corridor	Road; Passenger and freight railway; Port	Operational; In progress	Burundi; Democratic Republic of Congo; Rwanda; Tanzania; Uganda
14	CDH0001	Cairo-Dakar Corridor	Port	Operational	Mauritania
15	CGC0001	Central Growth Corridor Guinea	Passenger and freight railway	In progress; Operational	Benin; Guinea; Niger
16	CNC0001	Cotonou-Niamey Corridor	Port; Road	Operational; UPgrade	Benin
17	DAC0001	Dakar – Abidjan Corridor	Road	Planned	Côte d'Ivoire; Gambia; Guinea; Guinea-Bissau; Liberia; Senegal
18	DBC0001	Douala-Bangui Corridor	Road	Operational	Cameroon; Central African Republic
19	DBO0001	Dakar-Bamako-Ouagadougou –Niamey-N'Djamena Corridor	Port; Freight railway	Operational; In progress	Mali; Senegal
20	DDC0001	Dar es Salaam Development Corridor (or Uhuru Development Corridor)	Road; Passenger and freight railway; Pipeline (oil); Airport	Operational; Planned	Tanzania; Uganda; Zambia
21	DLC0001	Dakar-Lagos Corridor	Port	Operational	Côte d'Ivoire; Ghana; Guinea; Sierra Leone
22	DNC0001	Douala-N'djamena Corridor	Road; Passenger and freight railway	Operational; Planned	Cameroon; Chad
23	KCB0001	Kribi-Campo-Bata Corridor	Road; Port	In progress	Cameroon; Equatorial Guinea
24	KGR0001	Kigoma-Gitega Corridor	Passenger and freight railway	In progress	Burundi; Tanzania
25	KJA0001	Kampala-Juba-Addis Ababa-Djibouti Corridor	Road	Planned	Djibouti; Ethiopia; South Sudan; Uganda
26	KMI0001	King Mswati III International Corridor	Port	Operational	Eswatini
27	KMN0001	Kribi-Mbalam-Nabeba Corridor	Freight railway	In progress	Cameroon; Republic of the Congo

28	LAP0001	Lamu Port South Sudan Ethiopia Transport Corridor (LAPSSET)	Port; Road; Pipeline (oil); Passenger and freight railway; Airport; Resort city; Road	In progress; Operational; Planned	Ethiopia; Kenya; South Sudan
29	LBP0001	Libreville-Brazzaville-Pointe Noire Corridor (or Pointe Noire – Cabinda Corridor)	Road; Port	Upgrade; Operational	Gabon; Republic of the Congo
30	LCC0001	Cabinda Link	Road; Port	Operational	Angola; Democratic Republic of Congo
31	LIC0001	Lichinga Corridor	Road	Upgrade	Mozambique
32	LKJ0002	Lagos-Kano-Jibiya (LAKAJI) Agricultural Development Corridor	Road; Airport	Operational	Nigeria
33	LMP0001	Limpopo Corridor	Road	Planned	Mozambique
34	LOC0001	Lomé-Ouagadougou Corridor	Passenger and freight railway; Road; Port	In progress; Upgrade; Operational	Burkina Faso; Togo
35	LTT0001	Lake Tanganyika Transport Corridor	Waterway; Port	Operational	Burundi; Democratic Republic of Congo; Tanzania; Zambia
36	MAA0001	Massawa Corridor	Passenger and freight railway; Port	Planned; Operational	Eritrea; Ethiopia
37	MAC0001	Malange Corridor	Road	Operational	Angola; Democratic Republic of Congo
38	MDC0001	Maputo Development Corridor	Passenger and freight railway; Port; Road; Airport	Operational	Botswana; Eswatini; Mozambique; Namibia; South Africa
39	MDT0001	Maseru-Durban Transport Corridor	Road	Operational	Lesotho; South Africa
40	MDU0001	Manzini – Durban Corridor	Road	Operational	Eswatini; South Africa
41	MMC0001	South Madagascar Marine Corridor	Waterway	In progress	Madagascar
42	MTC0001	Mtwara Development Corridor	Port; Road; Freight railway; Waterway	In progress	Malawi; Tanzania
43	NAC0001	Nacala Corridor	Road; Passenger and freight railway; Port; Airport; Waterway	Operational; Planned; Operational	Malawi; Mozambique; Zambia
44	NBC0001	Namibe Corridor	Road	Operational	Angola
45	NEL0001	Nile Equatorial Lakes Subsidiary Action Program (NELSAP) Electricity Line	Electricity transmission	In progress	Burundi; Kenya; Tanzania; Democratic Republic of Congo; Rwanda; Uganda
46	NGC0001	Northern Growth Corridor Guinea	Freight railway; Port	Upgrade; Operational	Guinea
47	NIB0001	The Nimba-Buchanan Corridor	Freight railway	Operational	Guinea
48	NRC0001	Northern Corridor (or Trans Congo Corridor)	Road; Port; Freight railway; Airport; Dry port; Road	Operational; In progress	Burundi; Central Africa Republic; Democratic Republic of Congo; Kenya; Rwanda; South Sudan; Uganda
49	NSC0001	North-South Corridor	Passenger and freight railway; Pipeline (water); Electricity transmission; Airport; Port; Road; Passenger and freight railway	Planned	Botswana; Burundi; Democratic Republic of Congo; Egypt; Ethiopia; Kenya; Malawi; Mozambique; Rwanda; Sudan; South Africa; South Sudan; Tanzania; Zambia; Zimbabwe
50	OBN0001	Ouesso-Bangui-N'djamena Corridor	Road	In progress	Central African Republic; Chad; Republic of the Congo
51	PDA0001	Praia-Dakar-Abidjan Corridor	Waterway	Operational	Cabo Verde; Senegal
52	PNC0001	Pointe Noire – Cabinda Corridor (or Libreville-Brazzaville-Pointe Noire Corridor)	Road; Passenger and freight railway; Port; Airport	Operational	Angola; Republic of the Congo
53	POM0001	Port of Mogadishu	Port	Operational	Somalia
54	POT0001	Port of Toamasina	Port	Operational	Madagascar
55	POV0001	Port of Victoria	Port	Operational	Seychelles
56	PSC0001	Port Sudan Corridor	Port; Road	Operational	Sudan; South Sudan

57	SGC0001	Southern Growth Corridor Guinea	Freight railway	In progress, Planned	Guinea
58	SGR0001	Standard Gauge Railway Corridor	Passenger and freight railway; Dry port; Industrial parks	Operational; On hold; Planned	Kenya; Uganda; Rwanda; South Sudan
59	SNC0001	Sirari North – South Corridor	Road	Upgrade	Kenya
60	TAH0001	Trans-African Highway 1 (or Cairo-Dakar Corridor; or Trans-Magreb Highway)	Road	In progress	Egypt; Libya; Tunisia; Algeria; Morocco; Mauritania; Senegal
61	TAH0002	Trans-African Highway 2 (or Trans-Sahara Highway Corridor)	Road	In progress	Algeria; Tunisia; Mali; Niger; Chad; Nigeria
62	TAH0003	Trans-African Highway 3	Road	Operational	Libya; Chad; Niger; Cameroon; Central African Republic; Republic of Congo; Democratic Republic of Congo; Angola; Namibia; South Africa
63	TAH0004	Trans-African Highway 4	Road	Operational	Egypt; Kenya; Sudan; Ethiopia; Zimbabwe; Botswana
64	TAH0005	Trans-African Highway 5 (or Dakar-Bamako-Ouagadougou – Niamey-N'Djamena Corridor)	Road	Operational	Mali; Senegal; Niger; Burkina Faso; Chad
65	TAH0006	Trans-African Highway 6 (Djibouti-N'Djamena Corridor)	Road	In progress	Djibouti; Ethiopia; Sudan; Chad
66	TAH0007	Trans-African Highway 7 (or Dakar Lagos Highway or Trans Coastal West African Highway)	Road	Operational	Nigeria; Benin; Togo; Ghana; Côte d'Ivoire; Liberia; Sierra Leone; Guinea; Guinea-Bissau; Senegal; The Gambia
67	TAH0008	Trans-African Highway 8	Road	Operational	Nigeria; Cameroon; Central African Republic; Democratic Republic of the Congo; Uganda; Kenya
68	TAH0009	Trans-African Highway 9 (or Beira Lobito Corridor; Lobito Corridor)	Road	Upgrade	Mozambique; Zimbabwe; Zambia; Malawi; Democratic Republic of Congo
69	TAP0001	Trans African Pipeline Project	Pipeline (water)	Planned	Mauritania; Senegal; Eritrea; Ethiopia; Burkina Faso; Niger; Chad; Sudan; Djibouti; Nigeria; Mali
70	TCC0001	Trans-Caprivi Corridor (TCC) (or Walvis Bay-Ndola-Lumbumbashi Development Corridor (WBNLDC))	Road; Passenger and freight railway; Port	Operational	Democratic Republic of the Congo; Namibia; Zambia; Zimbabwe
71	TCU0001	Trans-Cunene Corridor (TCuC)	Road	Operational	Namibia; Angola
72	TDC0001	Tanga Development Corridor	Passenger and freight railway; Port; Road; Airport	Operational	Tanzania
73	TGC0001	Trans-Gambia Corridor	Road	Upgrade	Gambia
74	TKC0001	Trans-Kalahari Corridor (TKC)	Road; Passenger and freight railway	Operational; Planned	Namibia; Botswana; South Africa
75	TMC0001	Trans-Maghreb Corridor	Passenger and freight railway; Road	In progress; Operational	Morocco; Algeria; Tunisia; Libya
76	TOC0001	Trans-Oranje Corridor	Road; Passenger and freight railway; Port	Operational; Upgrade	Namibia; South Africa
77	TUC0001	Tema-Ouagadougou Corridor	Road; Passenger and freight railway	Operational	Burkina Faso; Ghana; Mali; Niger
78	WAT0001	West Africa Power Transmission Corridor	Electricity transmission	Operational	Ghana; Guinea; Guinea Bissau; Gambia; Sierra Leone; Liberia; Côte d'Ivoire
79	ZMR0001	Zambia-Malawi-Mozambique Railway Corridor	Passenger and freight railway	Operational	Zambia; Malawi
